# Parental concerns about COVID-19 vaccine safety and hesitancy in Korea: implications for vaccine communication

**DOI:** 10.4178/epih.e2023004

**Published:** 2022-12-13

**Authors:** Hye-Kyung Cho, Hyunju Lee, Young June Choe, Shinkyeong Kim, Sujin Seo, Jiwon Moon, Eun Hwa Choi, Geun-Yong Kwon, Jee Yeon Shin, Sang-Yoon Choi, Mi Jin Jeong, Myoungsoon You

**Affiliations:** 1 Department of Pediatrics, Ewha Womans University Mokdong Hospital, Seoul, Korea; 2 Department of Pediatrics, Seoul National University Bundang Hospital, Seongnam, Korea; 3 Department of Pediatrics, Seoul National University College of Medicine, Seoul, Korea; 4 Department of Pediatrics, Korea University Anam Hospital, Seoul, Korea; 5 Department of Public Health Sciences, Graduate School of Public Health, Seoul National University, Seoul, Korea; 6 Division of Immunization, Korea Disease Control and Prevention Agency, Cheongju, Korea

**Keywords:** SARS-CoV-2, COVID-19 vaccines, Child, Adolescents

## Abstract

**OBJECTIVES:**

Vaccination is one of the most important strategies to contain the spread of coronavirus disease 2019 (COVID-19). Vaccination in children is dependent on their parents, making it important to understand parents’ awareness and attitudes toward vaccines in order to devise strategies to raise vaccination rates in children.

**METHODS:**

A web-based nationwide survey was conducted among Korean parents of 7-year-old to 18-year-old children in August 2021 to estimate parents’ intention to vaccinate their children against COVID-19 and identify key factors affecting parental acceptance and hesitancy through regression analysis.

**RESULTS:**

Approximately 56.4% (575/1,019) were willing to vaccinate their children against COVID-19. Contributing factors to COVID-19 vaccine hesitancy were being a mother (adjusted odds ratio [aOR], 0.36; 95% confidence interval [CI], 0.25 to 0.52), a lower education level (aOR, 0.83; 95% CI, 0.70 to 0.97), hesitancy to other childhood vaccines (aOR, 0.78; 95% CI, 0.64 to 0.96), and refusal to vaccinate themselves (aOR, 0.08; 95% CI, 0.02 to 0.20). Having older children (aOR, 1.20; 95% CI, 1.13 to 1.28), trusting the child’s doctor (aOR, 1.19; 95% CI, 1.07 to 1.32), positive perceptions of the COVID-19 vaccine’s effectiveness (aOR, 2.60; 95% CI, 1.90 to 3.57) and perceiving the COVID-19 vaccine as low-risk (aOR, 1.68; 95% CI, 1.27 to 2.24) were associated with COVID-19 vaccine acceptance. Concerns about adverse reactions were the most common cause of hesitancy.

**CONCLUSIONS:**

Providing parents with accurate and reliable information on vaccine effectiveness and safety is important to increase COVID-19 vaccine uptake in children. Differential or targeted approaches to parents according to gender, age, and their children’s age are necessary for effective communication about vaccination in children.

## GRAPHICAL ABSTRACT

[Fig f4-epih-45-e2023004]

## INTRODUCTION

Vaccination is one of the most important strategies to control the ongoing coronavirus disease 2019 (COVID-19) pandemic. After COVID-19 vaccination began in adults in December 2020, vaccination in children under 16 years of age began in May 2021. However, there have been issues regarding the COVID-19 vaccine in that it uses a new platform and was developed in a relatively short period of time. In children, COVID-19 infection is known to have a relatively mild clinical course, but there have been reports of children with serious conditions or death [1,2]. In addition, the need for children’s COVID-19 vaccination is growing due to the contribution of the pediatric population, which makes up approximately 15-20% of the total population, to the spread of the virus in the community and indirect damages to children caused by prolonged social distancing or school closure during the pandemic. Therefore, careful decisions and approaches are required for pediatric COVID-19 vaccination policies.

Some studies have been conducted in different countries on parental perceptions of children’s COVID-19 vaccination [3-8], finding that parental acceptance of COVID-19 vaccine for their children differs among countries and depends on factors such as parental age, geographic location, ethnicity, household income, acceptance of other childhood vaccines, perceived susceptibility to and risk of COVID-19, and perceived vaccine safety and effectiveness. Korean parents have historically had a very high acceptance of childhood vaccinations, resulting in high vaccination rates [9]. However, since the introduction of the COVID-19 vaccine, various issues regarding the safety of the vaccine have been raised, resulting in negative perceptions of the vaccine [10]. Given the lower parental acceptance of the COVID-19 vaccine for children compared to other childhood vaccines [11], it is necessary to further investigate factors related to parental willingness for their children to be vaccinated against COVID-19.

Higher vaccine coverage is essential for improving the effectiveness of vaccines. To establish vaccination policies and strategies for this purpose, strategic communication between those to be vaccinated and policymakers should be devised based on an understanding of parents’ attitudes toward the vaccine and its contributing factors. This study aimed to analyze parental intentions to vaccinate their children against COVID-19 in Korea and factors influencing their attitudes, and to identify points to consider for improving vaccine coverage.

## MATERIALS AND METHODS

### Study subjects and survey methods

The cross-sectional web-based survey was performed among Korean parents of 7-year-old to 18-year-old children (first to 12th grades) across the country from August 11 to August 24, 2021, when COVID-19 vaccines were available only among adults aged ≥ 50 years and those with high-risk occupations in Korea. The survey was distributed via e-mail to a nationwide panel through a survey company. The subjects were selected by a proportional allocation extraction method by region, gender, age, and their child’s school and grade in school; elementary school (first to sixth grades), middle school (seventh to ninth grades) and high school (10th to 12th grades). Data such as demographic factors, child’s factors, perceptions of COVID-19 and COVID-19 vaccine, and their intention to vaccinate themselves and their children were collected. If a parent had multiple children, he or she was asked to choose only 1 and respond only regarding that child. The study was conducted according to the Strengthening the Reporting of Observational Studies in Epidemiology (STROBE) guidelines for cross-sectional studies [12].

### Questionnaire development

The survey included questions based on previous surveys about perceptions of vaccination [5,6], with the development of multiple additional questions. The questions were organized into the following categories: demographic information of parents and children, parents’ intention to vaccinate themselves and their children against COVID-19, acceptance of past childhood vaccinations (including flu vaccines in the last 5 years), trust in the child’s doctors, the impact of COVID-19 on daily life, perceptions of the COVID-19 vaccine, and reasons affecting parents’ intention to vaccinate their children against COVID-19. The subjects’ residence was classified into Seoul, Gyeonggi Province, and Incheon as the metropolitan region, and other cities and provinces as the non-metropolitan region. Parental acceptance of vaccinating their child was classified into the following 3 categories: “very unlikely” and “less likely” were categorized as “negative,” “undecided” as “moderate,” and “likely” and “very likely” as “positive.” Those who reported “positive” acceptance were asked to choose 2 reasons why they were likely to vaccinate their child against COVID-19, and those who reported “moderate” and “negative” acceptance were prompted to choose 2 reasons why they were unlikely to vaccinate their child.

Regarding the expected impact of getting COVID-19, participants were asked to answer a question (“If your child gets COVID-19, how serious do you think the impact [damage or loss] will be on the child’s health and daily life?”), with options including “very serious,” “serious,” “neutral,” “not serious,” or “not a problem.” They were also asked to answer a question about the expected risk of getting COVID-19 (“How likely do you think your child is to contract COVID-19?”), with possible responses of “very unlikely,” “unlikely,” “likely,” or “very likely.” They were also asked to select any of the following experiences that they had with COVID-19 among “my family members or I have been infected,” “my family members or I have been isolated as close contacts,” “my child’s school (or academy) teacher or friend has been infected,” “my child’s school (or academy) teacher or friend has been quarantined as a close contact,” and/or “not applicable.”

Participants were asked to answer a question about parental behaviors toward seasonal influenza vaccine (“How have you been immunizing your child against seasonal influenza within the last 5 years?”), with options including “vaccinated every year,” “vaccinated 2‒3 times,” “vaccinated at least once,” or “never vaccinated” within 5 years or “can’t remember whether or not vaccinated.” Next, they were asked to answer a question about parents’ hesitancy to other childhood vaccines (“How much do you hesitate about other childhood vaccines in general?”), with the following possible options: “never hesitate,” “do not hesitate,” “do not know,” “hesitate,” or “strongly hesitate.” They were asked to choose an option on a scale from 0 (not trust him/her at all) to 10 (trust him/her very much) regarding their trust in their child’s primary care physician.

Participants were asked to answer a question about their intention to vaccinate themselves against COVID-19 (“Would you have yourself vaccinated against COVID-19 if available?”), with possible responses including “definitely,” “probably,” “probably not,” “never,” “I don’t know,” or “already done.” They were also asked to choose an answer (“strongly agree,” “agree,” “neither agree nor disagree,” “disagree,” or “strongly disagree”) for the following statements: “the COVID-19 vaccine is safe for me (perceived safety of COVID-19 vaccine),” “the COVID-19 vaccine is effective to prevent infection, serious illness, and death (perceived effectiveness of the vaccine),” and “the possibility that adverse reactions occur after COVID-19 vaccination is low (perceived risk of the vaccine).”

### Statistical analysis

Descriptive statistics (i.e., frequencies and percentages for categorical variables, mean and standard deviation [SD] for continuous variables) were performed for the independent variables. We then assessed the associations between parents’ intention to vaccinate their children against COVID-19 and the independent variables using the chi-square test and multivariate logistic regression, reporting adjusted odds ratios (aORs) with 95% confidence intervals (CIs).

The predictive variables included in the logistic regression were characteristics commonly associated with vaccination hesitancy from the previous literature. Cases with missing data were excluded from the analysis.

To assess the predictors of parental intention to vaccinate their children against COVID-19, the associations between the variables and parental intention were analyzed using multivariate logistic regression analysis with R version 4.1.1 (R Foundation for Statistical Computing, Vienna, Austria).

### Ethics statement

This survey was conducted with support from the Korea Disease Control and Prevention Agency to be utilized in developing comprehensive vaccination policies for children. This study was performed with approval from the Korea University Institutional Review Board (protocol No. 2021AN0402).

## RESULTS

### Demographic characteristics of participants

A total of 1,025 parents participated in the survey, although 6 participants were excluded due to incomplete responses. Among 1,019 parents who completed the survey, 57.6% of the respondents were women, and most of them were in their 40s. The area of residence (metropolitan and non-metropolitan region), gender, and children’s school level (elementary, middle, and high school) were evenly distributed. Other factors collected included the parent’s education, monthly household income, child’s health condition and flu shot vaccination rate (for at least 1 shot during the past 5 years). Socio-demographic factors of the parents and their children are presented in Table 1.

### COVID-19 vaccine acceptance and contributing factors

**Figure 1.**
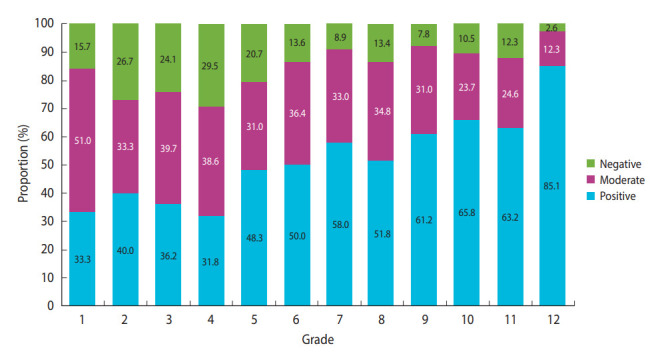
Coronavirus disease 2019 vaccine acceptance according to children’s grade in school.

The overall COVID-19 vaccine acceptance rate among parents of children in the first to 12th grades was 56.4%. Hesitancy was higher among mothers than among fathers. COVID-19 vaccine acceptance for children was lower in younger parents and parents with younger children (Table 2 and Figure 1). Low household income was also related to COVID-19 vaccine hesitancy. However, the expected impact on the child’s health and daily life from getting COVID-19 and the expected risk of the child contracting COVID-19 did not affect parental intentions to vaccinate their children. Experiences of being infected with or exposed to COVID-19, also did not affect parents’ intentions to vaccinate their child. Recent flu shot uptake for children in the past 5 years, which was high (92.2%), did not affect parental attitudes toward COVID-19 vaccination for their children, although respondents who showed hesitancy toward other childhood vaccines had lower acceptance of the COVID-19 vaccine than those who had not. Although 85.0% of the parents answered that they trusted their child’s doctor, those who did not trust their child’s doctor had lower acceptance of their child’s COVID-19 vaccine compared to those with trust in their child’s doctor. Parents who were themselves already vaccinated or were willing to be vaccinated showed higher acceptance of the vaccination for their children than parents who were not willing to get vaccinated themselves. Awareness of the effectiveness, safety, and risk of the COVID-19 vaccine was found to affect parental acceptance of the COVID-19 vaccine for their children.

**Figure 2.**
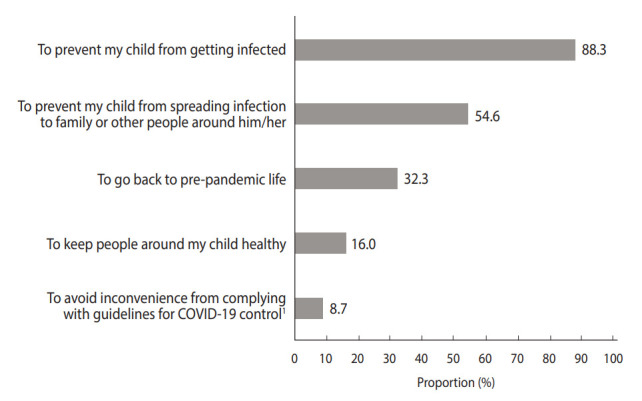
Reasons for parental coronavirus disease 2019 (COVID-19) vaccine acceptance for their children. Only those who showed acceptance of vaccination were asked to respond and choose 2 answers. ^1^e.g., Measuring body temperature, dining in partitions, and wearing masks.

**Figure 3.**
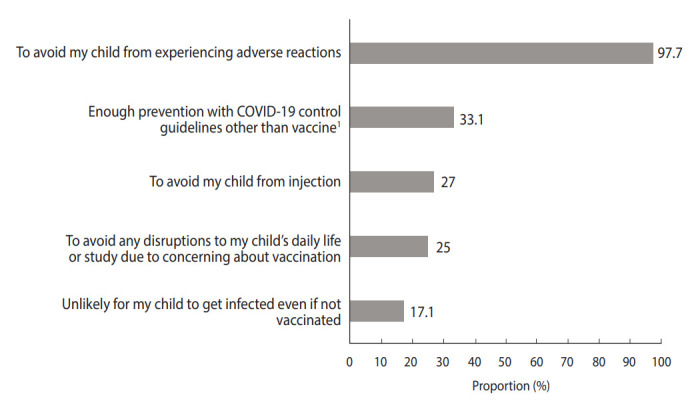
Reasons for parental coronavirus disease 2019 (COVID-19) vaccine hesitancy for their children. Only those who showed hesitancy toward vaccination were asked to respond and choose 2 answers. ^1^e.g., Measuring body temperature, dining in partitions, and wearing masks.

The most common reason to have their children vaccinated was “to prevent my child from getting infected,” followed by “to prevent those around you from COVID-19” (Figure 2). Meanwhile, the most common reason to hesitate about the COVID-19 vaccine for their children was “to avoid my child from experiencing adverse reactions,” followed by “adhering to other COVID-19 control policies is sufficient to prevent it” (Figure 3).

### Multivariate analysis of parents’ intention to vaccinate their children against COVID-19

The predictors of parents’ intention to vaccinate their children with COVID-19 vaccine were analyzed using multivariate binary logistic regression (Table 3). Being a mother (aOR, 0.36; 95% CI, 0.25 to 0.52), lower parental education levels (aOR, 0.83; 95% CI, 0.70 to 0.97), hesitancy toward other childhood vaccines (aOR, 0.78; 95% CI, 0.64 to 0.96) and a lack of parental willingness to be vaccinated themselves (aOR, 0.08; 95% CI, 0.02 to 0.20) were associated with lower parental intention to have their children vaccinated. Older children (aOR, 1.20; 95% CI, 1.13 to 1.28), trust in doctors (aOR, 1.19; 95% CI, 1.07 to 1.32), positive perceptions of the effectiveness of the COVID-19 vaccine (aOR, 2.60; 95% CI, 1.90 to 3.57), and considering the COVID-19 vaccine to be low-risk (aOR, 1.68; 95% CI, 1.27 to 2.24) were associated with parental acceptance of vaccinating their children.

## DISCUSSION

In this in-depth survey of parents of children 7-18 years of age, we analyzed parental acceptance of COVID-19 vaccination in children. This study helps to elucidate and better understand parental COVID-19 vaccine hesitancy for their children and its associated factors. According to the results, COVID-19 vaccine hesitancy was related to women gender, lower parental education, younger children, hesitancy toward other childhood vaccines, lack of trust in the child’s doctor, parental intention not to receive the vaccination themselves, and negative parental perceptions of the vaccine’s effectiveness and risks.

Parental intention to vaccinate their child against COVID-19 differs by country, the time of the survey, race/ethnicity, parental age, gender and educational level, children’s age, parental attitudes toward being vaccinated themselves, and experiences with COVID-19 [3,4,6,13-19]. In this study, factors affecting parental intention to vaccinate children included the age and gender of parents, the parents’ education level, the children’s age, and parental attitudes toward receiving the vaccination themselves, which is consistent with most studies from different countries [15,18,19]. Of note, parents of younger children were less likely to have their children vaccinated even if they had already been vaccinated or were willing to be vaccinated themselves. This finding suggests that parents seem to expect greater standards of safety profiles from vaccines for younger children. Parents of younger children may have more concern about potential long-term adverse effects, which was also the most important factor of vaccine hesitancy in many other studies [4,6,18-20]. This finding could be related to the widely known fact that younger adults or children with COVID-19 have mild symptoms and clinical courses [7]. Previous studies have reported that personal experience with COVID-19 affected parents’ attitudes toward vaccination [17,21,22]. A survey conducted in the United States showed that parental intention to vaccinate their child was associated with knowing someone who died of COVID-19 [15]. In this study, however, experiences of family members or the child’s friends or teachers being quarantined after being infected with or coming into contact with COVID-19 were not factors that affected the intention to vaccinate. It seems that the impact of experiences with COVID-19 on the intention to vaccinate may depend on the type (e.g., being infected or being quarantined) and severity (e.g., asymptomatic, severe illness, or death) of the experience.

As shown in other studies, parental perceptions of vaccine safety and adverse reactions were the most important factors associated with a positive intention to vaccinate in this study [23]. In addition, most parents answered that the reason for their hesitancy to have their children vaccinated against COVID-19 was “to avoid my child from experiencing adverse reactions.” Most studies have consistently shown that concerns about adverse reactions to the vaccine and unknown long-term adverse effects are the most influential factors for parental hesitancy toward the COVID-19 vaccine [13,15,18,19]. In particular, as the number of people who access information about vaccines through social media and internet messages increases, there are growing concerns regarding misinformation or mistrust about vaccines from unreliable sources [4,24-26]. This suggests that providing reliable information on vaccine safety should be considered as a top priority. Some studies have emphasized the role of physicians or health care providers in communication with parents about children’s COVID-19 vaccination [6] and others reinforced the need for health authorities to take the lead in providing knowledge about COVID-19 vaccine, along with transparent information related to adverse reactions associated with this vaccination [13,14].

In this cross-sectional survey, which was conducted in August 2021, when 41.6% of the total Korean population had received at least 1 dose of the vaccine (15.4% had completed the vaccination series), 56.4% of Korean parents reported acceptance of the COVID-19 vaccine for their children. Although studies on the intention of Korean parents to have their children vaccinated against COVID-19 are very limited, a study showed that 64.2% of the parents intended to vaccinate their children, which was higher than that in this study [16]. The higher acceptance rate observed in the previous study might be explained by the fact that it was hospital-based and the participants were likely to be at an elevated risk for severe COVID-19. In another Korean study that was performed in December 2021, when most adults had completed their vaccination series in Korea, 34.2% of parents reported acceptance of the COVID-19 vaccine for their child [27]. Therefore, acceptance rates may fluctuate across different time points and epidemic waves according to parents’ experiences and information about adverse reactions or breakthrough infections, which is consistent with other survey-based research performed before and after the implementation of COVID vaccination [18]. Therefore, it is necessary to continue monitoring changes in parental intentions to improve vaccination rates.

In Korea, the mRNA vaccine for children aged 12-15 years and those aged 5-11 years has been available since November 1, 2021 (October 18, 2021 for those aged 16-17 years) and March 31, 2022, respectively [28,29]. As of December 31, 2021, 74.0% of children aged 12-17 years in Korea had received at least one dose of the vaccine [30], which was higher than the percentage of parents who intended to vaccinate their children in this survey (56.0% in 12-15 year-olds and 71.3% in ≥ 16 year-olds). This might have been because more safety information was collected from Korea and other countries for about 4 months after the survey. The increase in vaccination among friends and family members may have contributed to the increased uptake of the COVID-19 vaccine in children 12-17 years of age. Furthermore, in early December 2021, the Korean government announced that it would apply the quarantine pass for entrance to private academies and study rooms starting in March 2022, which seems to have had a major impact on raising the vaccination rate in this age group. The vaccination rate has risen during the winter break as children and parents may prefer to avoid disruptions to schoolwork and prepare for the new school year, which starts in March. However, as of May 9, 2022, the vaccination rate for children aged 5-11 in Korea was 1.3%, much lower than the rate that would be expected from this study (38.4% in ≤ 11-year-olds) as well as than that of other countries (e.g., 20% in Germany, 35% in the United States, and 57% in Spain) [31,32]. This may have resulted from the relatively low severity of COVID-19 in young children and the waning vaccine effectiveness during the Omicron surge with rising breakthrough infections since the end of January 2022 in Korea. However, the significant difference in the vaccination rate between children aged 12-17 years and those aged 5-11 years may also reflect a disparity in parents’ perceptions of vaccine-related adverse reactions. As seen in our regression analysis, parents’ perception of the vaccine safety did not differ between the acceptance group and the hesitancy group, but concerns about adverse reactions had a negative effect on parents’ intention to vaccinate their children. This finding has been consistently observed in other studies as well [18,19], which means that concerns about adverse reactions do not simply reflect misinformation that can be solved by educating parents about vaccine safety; instead, some hesitancy originates intuitively from persistent psychological traits [33]. Since this psychological reactance is likely to affect parents of younger children, makes communication with physicians less effective, and results in hesitancy toward vaccination [34-36], alternative communication strategies tailored to this population are required.

This study has some limitations. Because this study was conducted among the parents of elementary, middle, and high school students (7-18 year-olds) in Korea, the parents of 12th graders, approximately 95% of whom had already been vaccinated at the time of the survey, were included. As a result, overall parental willingness may have been overestimated in this study compared to the actual percentage of parents who intended to have their children vaccinated.

Despite these limitations, this study provides important information on parents’ perceptions of the COVID-19 vaccine, their intention to vaccinate their children, and factors related to their attitudes. These findings help to devise strategies to overcome hesitancy toward the vaccine and improve vaccine coverage in children. This study also has the advantage that the subjects were enrolled nationwide and evenly by region and age, making it possible to conduct a survey representing parents across the country.

Approximately half of Korean parents intended to vaccinate their children against COVID-19, and higher hesitancy was shown in younger children’s parents. Parents’ concerns about experiencing adverse reactions were the largest factor affecting awareness and attitudes toward COVID-19 vaccination for their children, which is consistent with previous studies. Strategies to provide parents with accurate and reliable vaccine information will be important to increase the uptake of COVID-19 vaccination in children. Differential or targeted approaches to parents according to gender, age, and the age of their children may also be necessary for effective communication to mitigate parents’ concerns about COVID-19 vaccination in children.

## Figures and Tables

**Figure f4-epih-45-e2023004:**
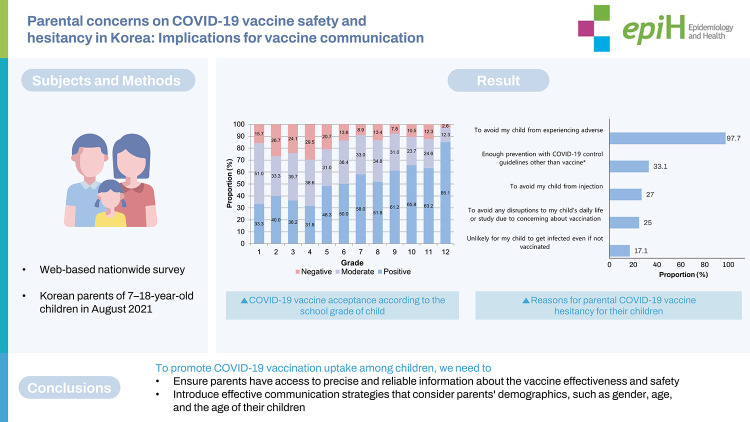


**Table 1.** Demographic characteristics of participants

Characteristics	n (%)
Parental factors	
Gender	
Men	432 (42.4)
Women	587 (57.6)
Age (yr)	
30-39	126 (12.3)
40-49	762 (74.8)
≥50	131 (12.9)
Residence	
Metropolitan region	506 (49.8)
Non-metropolitan region	513 (50.2)
Education	
High school and below	134 (13.1)
College and above	885 (86.9)
Monthly household income (104 KRW)	
≤200	37 (3.6)
200-400	252 (24.7)
400-600	377 (37.1)
≥600	353 (34.6)
Child factors	
Child’s gender	
Men	520 (51.0)
Women	499 (49.0)
Child’s age (yr)	
≤11	271 (26.6)
12-15	406 (39.8)
≥16	342 (33.6)
School grade	
1st	51 (5.0)
2nd	60 (5.8)
3rd	58 (5.7)
4th	44 (4.3)
5th	58 (5.7)
6th	66 (6.5)
7th	112 (11.0)
8th	112 (11.0)
9th	116 (11.4)
10th	114 (11.2)
11th	114 (11.2)
12th	114 (11.2)
Child’s health condition	
Bad	18 (1.8)
Moderate	244 (23.9)
Good	757 (74.3)
Flu shot uptake in the last 5 yr	
Yes	940 (92.2)
No/Don’t know (uncertain)	79 (7.8)

KRW, Korean won.

**Table 2.** Univariate analysis of factors contributing to parental COVID-19 vaccine acceptance

Variables			Acceptance	Hesitancy	p-value
Overall			575 (56.4)	444 (43.6)	
Parental factors					
	Gender				<0.001
		Women	265 (45.1)	322 (54.9)	
		Men	310 (71.8)	122 (28.2)	
	Age (yr)				<0.001
		30-39	48 (38.1)	78 (61.9)	
		40-49	428 (56.2)	334 (43.8)	
		≥50	99 (75.6)	32 (24.4)	
	Residence				0.211
		Metropolitan region	296 (58.4)	211 (41.6)	
		Non-metropolitan region	279 (54.5)	233 (45.5)	
	Education				0.656
		High school and below	78 (58.2)	56 (41.8)	
		College and above	497 (56.2)	388 (43.8)	
	Monthly household income (10^4^ KRW)				<0.001
		≤200	15 (40.5)	22 (59.5)	
		200-400	118 (46.8)	134 (53.2)	
		400-600	212 (56.2)	165 (43.8)	
		≥600	230 (65.2)	123 (34.8)	
Child factors					
	Child’s gender				0.576
		Women	286 (57.3)	213 (42.7)	
		Men	289 (55.6)	231 (44.4)	
	Child’s age (yr)				<0.001
		≤11	104 (38.4)	167 (61.6)	
		12-15	227 (56.0)	179 (44.1)	
		≥16	244 (71.3)	98 (28.7)	
	Child’s health condition				0.431
		Bad	10 (55.6)	8 (44.4)	
		Moderate	129 (52.9)	115 (47.1)	
		Good	436 (57.6)	321 (42.4)	
Expected impact and risk					
	Expected impact on child’s health and daily life from getting COVID-19				0.826
		Not serious	30 (60.0)	20 (40.0)	
		Moderate	76 (57.6)	56 (42.4)	
		Serious	469 (56.0)	368 (44.0)	
	Expected risk of getting COVID-19				0.265
		Low	269 (59.3)	185 (40.7)	
		Moderate	257 (54.2)	217 (45.8)	
		High	49 (53.8)	42 (46.2)	
COVID-19 experience					0.480
	COVID-19 infection and exposure of family members, child’s teachers, or friends		311 (55.4)	250 (44.6)	
	No infection or exposure		264 (57.6)	194 (42.4)	
Attitude to other childhood immunization and child’s doctor					
	Flu shot uptake in the last 5 yr				0.398
		Yes	534 (56.8)	406 (43.2)	
		No/Uncertain	41 (51.9)	38 (48.1)	
	Hesitancy toward other childhood vaccines				<0.001
		Not hesitant	457 (64.0)	257 (36.0)	
		Hesitant/Uncertain	118 (38.7)	187 (61.3)	
	Trust in the child’s doctor				<0.001
		No trust (scale 0-5)	52 (33.8)	102 (66.2)	
		Trust (scale 6-10)	523 (60.5)	342 (39.5)	
Perceptions of COVID-19 vaccination themselves					
	Intention to vaccinate themselves				<0.001
		Yes	508 (62.6)	303 (37.4)	
		No/Uncertain	4 (4.7)	81 (95.3)	
		Already vaccinated	63 (51.2)	60 (48.8)	
Perceptions of the COVID-19 vaccine					
	The COVID-19 vaccine is safe				<0.001
		Agree	260 (81.0)	61 (19.0)	
		Neutral	268 (49.5)	273 (50.5)	
		Disagree	47 (29.9)	110 (70.1)	
	The COVID-19 vaccine is effective				<0.001
		Agree	438 (73.9)	155 (26.1)	
		Neutral	128 (34.8)	240 (65.2)	
		Disagree	9 (15.5)	49 (84.5)	
	The risk of the COVID-19 vaccine is low				<0.001
		Agree	310 (50.0)	309 (50.0)	
		Neutral	234 (63.9)	132 (36.1)	
		Disagree	31 (91.2)	3 (8.8)	

Values are presented as number (%). COVID-19, coronavirus disease 2019; KRW, Korean won.

**Table 3.** Multivariate analysis of factors affecting parental intention to have their children vaccinated against COVID-19

Variables	aOR (95% CI)	p-value
Gender, women (ref: men)	0.36 (0.25, 0.52)	<0.001
Age	1.04 (0.99. 1.09)	0.069
Residence non-metropolitan (ref: metropolitan)	0.94 (0.68, 1.28)	0.678
Education	0.83 (0.70, 0.97)	0.023
Monthly household income	1.08 (0.99, 1.18)	0.054
Child’s gender, women (ref: men)	1.04 (0.76, 1.42)	0.803
Child’s age	1.20 (1.13, 1.28)	<0.001
Child’s health condition	1.11 (0.89, 1.39)	0.361
Expected impact on child’s health and daily life from getting COVID-19	1.17 (0.95, 1.44)	0.147
Expected risk of getting COVID-19	1.01 (0.83, 1.24)	0.891
COVID-19 experiences, yes (ref: no)^1^	0.90 (0.65, 1.24)	0.509
Flu shot uptake in the last 5 yr, yes (ref: no)	1.04 (0.57, 1.90)	0.902
Hesitancy toward other childhood vaccines	0.78 (0.64, 0.96)	0.017
Trust in the child’s doctor	1.19 (1.07, 1.32)	0.001
Parental intention to be vaccinated themselves, hesitancy (ref: accept)	0.08 (0.02, 0.20)	<0.001
Perceived safety of the COVID-19 vaccine	1.06 (0.78, 1.43)	0.721
Perceived effectiveness of the COVID-19 vaccine	2.60 (1.90, 3.57)	<0.001
Perceived risk of the COVID-19 vaccine, low	1.68 (1.27, 2.24)	<0.001

All variables except variables with reference categories were included in the regression analysis as continuous variables. COVID-19, coronavirus disease 2019; aOR, adjusted odds ratio; CI, confidence interval; ref, reference value.

1 COVID-19 infection and exposure of family members, child’s teachers or friends, yes (ref = no).
